# MarpoDB: An Open Registry for *Marchantia Polymorpha* Genetic Parts

**DOI:** 10.1093/pcp/pcw201

**Published:** 2017-01-27

**Authors:** Mihails Delmans, Bernardo Pollak, Jim Haseloff

**Affiliations:** Department of Plant Sciences, University of Cambridge, Downing Street, Cambridge, CB2 3EA, UK

**Keywords:** Genetic parts, genetic databases, *Marchantia polymorpha*, open source database, plant synthetic biology

## Abstract

*Marchantia polymorpha* is an extant relative of the earliest terrestrial plants and has attracted a substantial interest as a model organism for evolutionary and developmental studies. Given its relatively simple genome, compact gene families, simple morphology, ease of propagation and transformation, *M. polymorpha* is becoming a promising platform for plant synthetic biology. Modular genetic parts have been essential for development of synthetic biology approaches, so we sought to design an engineering oriented database for *M. polymorpha* genetic parts where each gene is a stand-alone functional unit. MarpoDB is a database of *M. polymorpha* genes and genetic parts, which is tailored to become an integral tool for a synthetic biology workflow. Among its features are precompiled cross-database querying to InterPro, Pfam signatures and non-redundant *Viridiplantae* BLAST annotations; BLAST querying to *M. polymorpha* genes; sequence export in GenBank format; recoding of sequences to the common syntax for type IIS assembly and exchange of DNA parts; and a minimalistic, intuitive and interactive user interface for gene models and sequence exploration. Furthermore, we have implemented user input to encourage feedback, collaboration and exchange between the MarpoDB community. MarpoDB source-code is released on GitHub to promote development of computational tools for synthetic biology.

## Introduction

Since the mid 1960s, development of novel sequencing and computational methods enabled the generation of genetic and protein sequence databases (Eck & Dayhoff 1966). As sequencing technologies improved, ambitious projects for sequencing whole genomes led to the creation of comprehensive genomic databases integrating not only DNA, RNA and protein sequences, but also corresponding publications, biological reference materials, genetic and physical markers, as well as, experimental data (Ashburner and Drysdale, 1994; Huala et al. 2001). Largely referred to as genomic resources, these databases have become the cornerstones for biological research. However, in pursuit of comprehensiveness, they suffer from information overflow, and become overly complicated for new types of specific applications, which could benefit from simpler user interfaces and different database models.

Synthetic biology is a new field of research, which aims to engineer biological functions and has generated interest in the plant community (Medford & Prasad 2014, Liu & Stewart 2015, Nemhauser & Torii 2016). It applies concepts of modularity to genetic sequences by treating functional elements, such as proximal and core promoters elements or coding sequences, as independent parts that can be mixed and matched in order to engineer novel biological functions. This modular approach extends from parts definitions to higher levels of abstraction in a hierarchical fashion: parts compose devices, the minimal functional elements; devices are arranged into circuits and circuits implemented into systems (Endy 2005, Andrianantoandro et al. 2006, Purnick & Weiss 2009, Federici et al. 2013). Adopting this modular approach allows simpler conceptual frameworks for experimentation and gives improved access for non biologists. Furthermore, parts can be tested in different genetic contexts and characterized for their performance to develop predictive computational models of biological circuits, such as logic gates (Nielsen et al. 2016). In order to store definitions of parts and higher order genetic components, synthetic biology requires DNA sequence databases, which are significantly different in design, compared to conventional genomic resources.

Attempts to collect and standardize descriptions of genetic components have been made, for example, by the Registry for Standard Biological Parts (http://parts.igem.org), which gained its popularity through the international Genetically Engineered Machine Competition (iGEM). Definitions of genetic parts, introduced by the registry, have enabled the development of standardized DNA assembly systems (Shetty et al. 2008), automation of DNA construction, and higher-level design of genetic circuits. Recently, a common syntax for type IIS assembly of plant DNA parts has been adopted by a large part of the plant synthetic biology community, which significantly simplifies design, assembly and sharing of DNA parts (Patron et al. 2015). The syntax defines 12 fusions sites and other required plasmid features, such as the absence of BsaI restriction sites, for using sequences in type IIS assembly systems. Furthermore, iGEM has embraced this common syntax; and plant genetic modules, Phytobricks, are currently being accepted to the registry: http://2016.igem.org/Resources/Plant_Synthetic_Biology/PhytoBricks. One drawback of the Registry for Standard Biological Parts is that the sequences of genetic components were extracted in the pre-genomic era, and hence are conceptually separated from the original genetic context. Since it is now possible to exploit genomic resources as a source for novel genetic parts, a major advance would be to incorporate part definitions directly into the dataset, maintaining and tracking the relationships between the endogenous sequences and the engineered parts.

*Marchantia polymorpha* is a complex thalloid liverwort, and is becoming an emerging model for plant biology. *M. polymorpha* is dioecious and has a dominant haploid gametophytic lifestyle (during the gametophytic phase, male plants lack the X chromosome and female plants lack the Y chromosome). It produces vegetative propagules, but also reproduces sexually through spores, which develop from single cells in culture. The genome of *M. polymorpha* Tak-1 strain has been sequenced and made available as an early release through Phytozome (Goodstein et al. 2012) by the *M. polymorpha* community sequencing effort (https://phytozome.jgi.doe.gov/pz/portal.html#!info?alias=Org_Mpolymorpha_er); and presents an excellent opportunity to uncover novel genetic parts and functions. Preliminary reports show that gene families in *M. polymorpha* show remarkable lack of redundancy compared to higher plants such as *Arabidopsis thaliana* (Sasaki et al. 2007, Flores-Sandoval et al. 2015, Kato et al. 2015). Furthermore, an extended array of techniques (Ishizaki et al. 2016) and molecular tools are available for *M. polymorpha*, including transformation methods (Chiyoda et al. 2008, Ishizaki et al. 2008, Tsuboyama & Kodama 2014, Tsuboyama-Tanaka & Kodama 2015), vectors and markers (Ishizaki et al. 2015), homologous recombination (Ishizaki et al. 2013) and Cas9-mutagenesis (Sugano et al. 2014). All of the above make *M. polymorpha* an attractive candidate as a basal plant chassis for synthetic biology.

Complete genomic sequences are useful for purposes where genome structure is relevant, for example, chromosome conformation capture, comparative genomics and physical genetic mapping. Although high-order chromatin organization has been acknowledged to play an important role in the regulation of genetic programs (Rosa & Shaw 2013), at the level of engineering genetic devices, we need to define functional elements that show robust behavior decoupled from inherent complexities of higher order control. Therefore, functional genetic regions, i.e. coding sequences and their proximal regulatory elements, are sufficient for the purpose of engineering synthetic genes. Then, self-contained genetic modules can be abstracted from the genome and intergenic sequences omitted. To explore this idea, we have established the *M. polymorpha* Cam-1 strain, which is easily induced for sexual reproduction and propagation, performed next-generation sequencing, and developed a new gene-centric database framework for synthetic biology.

Here we present MarpoDB, an open registry for efficient identification of putative *M. polymorpha* genetic parts. While the database is based on the Cam-1 strain, it integrates cross-referencing to the *M. polymorpha* Tak-1 community genome dataset (http://genome.jgi.doe.gov/Marpha/Marpha.info.html). MarpoDB includes a set of computational tools for genetic engineering: mining for *M. polymorpha* genes by querying from precompiled BLAST results (Altschul et al. 1990) and InterPro signatures (Mitchell et al. 2015); performing of BLAST search queries against MarpoDB; recoding of parts into the common syntax for type IIS assembly; visualization of gene models and DNA sequence through a minimalistic, intuitive and interactive user interface; and direct sequence export in GenBank format. Moreover, we included information regarding community activity in the form of top favored genes and a geographical map of MarpoDB users. Although MarpoDB is a *M. polymorpha* database, the database model is agnostic to the source of genetic sequence. Hence, it may be reused for other species or with a collection of DNA sequences such as a plasmid library. Source code for *MarpoDB,* along with setup instructions have been uploaded to GitHub to encourage implementation of the database framework for other datasets and promote distributed development. Finally, we used MarpoDB to mine for Alpha tubulin-like, RuBisCO small subunit-like and Ubiquitin-like promoter elements; and tested them by fluorescent promoter fusion reporters. In addition, we used MarpoDB to perform a batch retrieval of potential *M. polymorpha* transcription factor promoters elements for high-throughput DNA synthesis.

## Results

### Data preparation

**Fig. 1:**
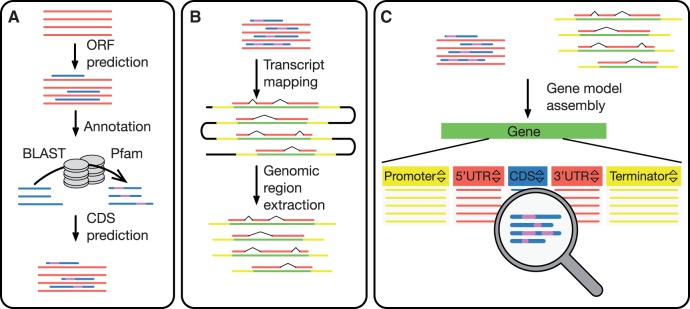
Data preparation and assembly. Red stripes denote the transcripts; blue stripes denote CDSs and corresponding proteins; purple stripes denote Pfam and BLAST annotation; yellow stripes denote 3 Kb regions which flank transcript mapping sites on a genome; black stripes denote a genome sequence. (A) Prediction of CDSs and annotation: The longest ORFs were predicted using TransDecoder software. Corresponding protein sequences were annotated using BLAST to a Uniprot protein database and Pfam signature analysis. (B) Extraction of genomic sequences: Transcripts, which passed annotation and CDS prediction analysis, were mapped to the genome assembly using Splign software. Genomic regions, which corresponded to the transcript mapping sites, were extracted together with 3 Kb flanking regions, to account for potential promoter sequences. (C) Assembly of the gene models: Information about transcript mapping sites, CDSs, protein annotations, and extracted genomic regions were assembled into a gene model, where each gene is an assembly of a promoter, transcript and terminator. Every gene stores a relative genomic location, based on its mapping site.

In order to generate data for MarpoDB, we prepared DNA and RNA extracts from tissue of *M. polymorpha* Cam-1 strain (male plants) and subjected it to Illumina DNA sequencing, which provided 72.52 M paired-end reads, with approximately 50X coverage. A *de novo* genome assembly was conducted using Meraculous assembly (Chapman et al. 2011), providing 38,333 contigs with an N50 of 34.2 Kb and 15,703 scaffolds with an N50 of 44.8 Kb, yielding a total of 195.3 Mb of assembled nucleotide pairs. RNA sequencing produced 193.8 M reads, which were used to generate a *de novo* transcriptome assembly via Bridger assembler (Chang et al. 2015). The assembly produced 93,315 isoforms that were further subjected to ORF prediction and mapping to the DNA assembly (Fig. 1A, B). After filtering out unmapped and non-coding transcripts, we were left with 36,337 potential protein sequences distributed across 18,934 potential coding isoforms. Further, the potential protein sequences were annotated using BLAST to Uniprot Viridiplantae protein sequences (The Uniprot Consortium 2014) and InterPro analysis. Protein family (Pfam) hits, originated from the InterPro analysis, were filtered by gathering thresholds (Finn et al. 2016); protein BLAST results were filtered by a minimal identity of 35% and minimal coverage per subject of 20%. Next, the isoforms we filtered, retaining only those, which had at least one predicted CDS and one hit against Pfam or Uniprot databases.

### Database assembly

As a result of the data preparation step, we collected information about every isoform location relative to the Cam-1 genome, every predicted CDS location relative to the corresponding transcript, as well as locations of BLAST and Pfam hits relative to the corresponding CDS. We used the following approach to compile this information into MarpoDB: First, we assigned isoforms with overlapping genomic locations to a same locus; then, we extracted the genomic sequence for each locus, preserving 3 Kb flanks as potential promoter elements and terminator sequences; lastly, we split loci sequences into promoter elements, 5′UTR, predicted CDS, 3′UTR and terminator sequences for each mapped isoform. 5′UTRs were defined from the mapped transcriptional start site to the start of the putative ORF, and 3′UTRs from the end of the putative ORF to the end of the transcript. For loci with several mapped isoforms the splitting was made separately for each isoform.

To frame the obtained data into a genetic part registry format, we applied the following conceptual schema, which laid out the foundation for the database model (see Fig. 1C). The key elements of the MarpoDB are genes. As noted previously, we deliberately did not include genome level into the model, so each gene is considered as an independent entity. Furthermore, the gene entries do not store the DNA sequence; instead, the sequence is stored for every genetic part, and the gene is represented as an assembly of the parts in a strict order: promoter—5′UTR—CDS—3′UTR—terminator. This approach allows us to represent gene sequences in a modular fashion, maintaining the endogenous context while attributing new definitions directly onto the dataset. In addition, we store coordinates of exons for UTRs and CDSs. This allows us to retain the original genomic sequence, whilst being able to recover the spliced sequence for the transcribed parts. Although sequences for the genome assembly are not stored, each gene is associated with one loci, which records the original genomic coordinates. Finally, we store a collection of BLAST hits against UniprotKB and Pfam annotation for each CDS. Each hit entry stores keywords, e.g. gene name, protein name, Uniprot ID, Pfam accession; which allows mining for *M. polymorpha* genes by matching them to a user query.

### Web interface

**Fig. 2:**
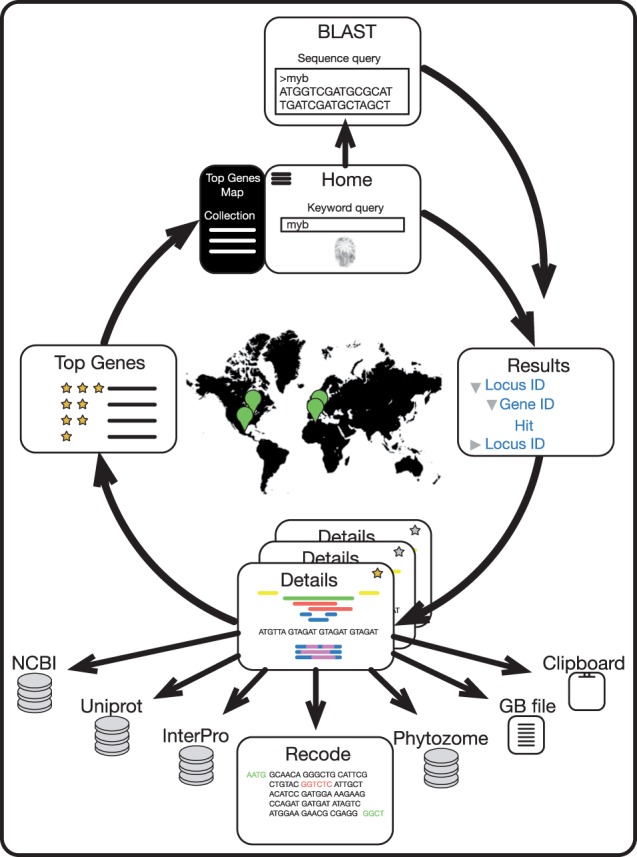
MarpoDB workflow. Top: Upon query submission at the Home or BLAST page, a user is presented with a table of results (right), where each row corresponds to a gene entry, containing a query keyword. Bottom: Selecting one of the results from the list opens up the Details page, which contains the gene model chart, and sequence viewer along with BLAST and Pfam annotations. From the Details page, the user can proceed with a BLAST search of the selected sequence in the NCBI database; export the gene model in GenBank format; or copy the selected sequence into the clipboard. Alternatively the user may proceed to the Recode page for adapting the selected sequence for type IIS assembly. In addition, the user can ‘star’ a gene, which will add it to the user’s personal collection, as well as increase the gene score in the global top (left).

MarpoDB is accessible via a web interface at http://marpodb.io. The layout is composed of a navigation menu at the top, current page title and the main content section. The navigation menu includes links to the ‘About’, ‘Registration’ and ‘Help’ pages, login form, as well as a sliding sidebar, which contains community related information: top genes and a geographical community map. When logged in, the sliding sidebar also shows a collection of user-saved genes for a quick access. The MarpoDB workflow is depicted in Fig. 2 and a detailed explanation of the website sections is presented below.

#### Home

The home page features a search form, which invites users to query the database by homologous protein/gene name, protein family domain name or accession, MarpoDB ID, or Phytozome ID. In addition, the search form contains a link to the ‘BLAST to MarpoDB’ section. The footer includes authors’ contact information and references to JavaScript libraries, used for the application front-end.

#### Results

After the query is processed, a user is presented with an interactive table of results. Each row corresponds to a locus, which has at least one MarpoDB gene with an annotation(s) that matches the user query. The table is sorted by an E-value, and displays relevant information for each hit, e.g. Pfam accession and Pfam description for a Pfam hit; or gene name and protein name for a BLAST hit. In addition, each row can be expanded to display information about the gene that produced the hit. The user proceeds by clicking on the gene ID or locus ID, which brings them to the ‘Details’ page.

#### Details

The details page contains a color-coded interactive gene model for isoforms in the selected locus. Clicking on a feature highlights the sequence, which is presented in the sequence viewer. Below the sequence viewer is detailed information for the selected CDS. Selecting another CDS will refresh details page, and load the corresponding information.

The sequence viewer displays the genomic sequence, and features a search bar, which supports regular expressions. Below the sequence viewer, there are buttons for performing BLAST of the highlighted sequence to the NCBI database (Geer et al. 2010), exporting the annotated sequence to GenBank file format, copying the highlighted sequence to clipboard or recoding the sequence into a type IIS part with Bsal restriction sites removed and common syntax overhangs appended. Further below, there are two clickable tabs with InterPro and protein BLAST annotations. The InterPro tab contains a domesticated HTML-formatted output of InterPro Scan, which illustrates the CDS with predicted protein domains, signatures and associated gene ontology terms. Hovering over the different predictions displays a tooltip with detailed information about the features and links to the InterPro site with detailed information about the domain. The protein BLAST tab shows a gene illustration with information about the hit coverage, identity of the alignment, gene name, organism of origin and E-value.

When present, an alias with a link to a closest homolog in the community genomic resource is provided below the title. If logged in, a star appears next to the title, which allows saving the gene in the user’s account favorite genes, accessible through the sliding menu.

#### Recode

The recode page presents a view of the original sequence, with BsaI and SapI restriction sites highlighted. If the selected sequence is a CDS, a selector field allows defining what type of fusion (N-terminal, C-terminal or no fusion) should the sequence be recoded to. This specifies the types of overhangs to be added according to the common syntax standard. Once the recode button is pressed, the sequence viewer presents the recoded sequence with the new BsaI sites, overhangs and old restriction sites highlighted in different colors. Lastly, pressing the GenBank file button exports the annotated sequence file.

#### BLAST to MarpoDB

Accessing the BLAST to *MarpoDB* page reveals a text area, where either raw or FASTA-formatted sequence can be entered. Below, a type of a BLAST program can be selected, as well as, E-value cutoff and substitution matrix. Once the BLAST button is pressed, a BLAST result table is generated, which displays detailed information about the quality of the hit, sequence alignment, as well as a link to ‘Details’ page for the corresponding gene and locus.

#### About

The About page is accessible through the navigation bar or the home page. Here, there is a general description of *MarpoDB*, technical details and a disclaimer.

#### Registration

The registration page is accessible through the navigation menu only through the home page, and contains a registration form.

#### Help

The Help page features a pictorial representation of MarpoDB workflow along with a brief description of each page and how to interact with the user interface.

### Validation and transcription factors promoter library design

**Fig. 3:**
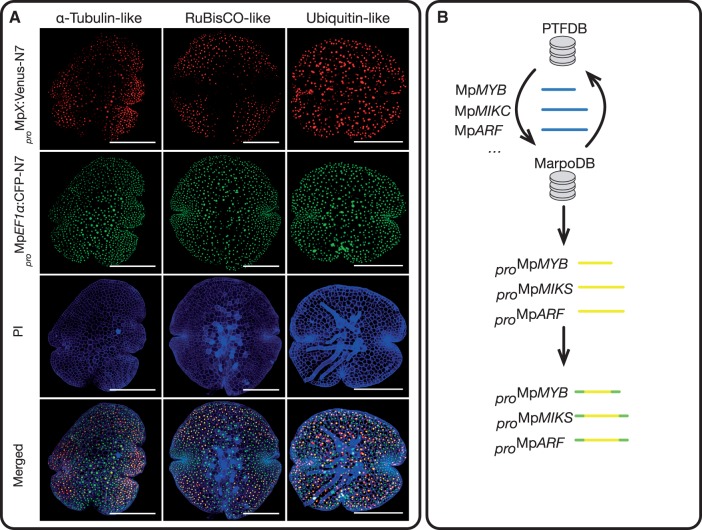
Applications of MarpoDB. (A) Confocal images for three different promoter elements predictions tested by promoters fusions reporters. Plasmids contained a _pro_MpEF1a:mTurquoise2-N7 cassette and promoters were introduced upstream of a Venus-N7 sequence. Gemmae from transformed plants were used for confocal imaging. Scale bars represent 250 microns. (B) Batch retrieval of *M. polymorpha* transcription factors’ promoters. CDS sequences of all MarpoDB genes were extracted and queried against the Plant Transcription Factor Database. IDs of CDSs with transcription factor prediction were then queried back to MarpoDB to extract the corresponding promoter elements’ sequences. Finally, sequences were recoded to match the standard syntax for type IIS assembly.

To validate if core element promoters are sufficient to capture promoter activity we extracted 2 kb promoter elements together with 5′UTRs for RuBisCO small subunit-like, Alpha tubulin-like and Ubiquitin-like candidates from our dataset. We cloned the promoters into pGreen derived vectors (Hellens et al. 2000) upstream of a Venus-N7 gene, together with a *_pro_*Mp*EF1a*:mTurquoise2-N7 cassette, which was used as a constitutive reference channel. When transformed and examined under a confocal microscope, *M. polymorpha* gemmae exhibited a distinct expression pattern of Venus for each of the selected promoters: Alpha tubulin-like promoter element fusion was expressed preferentially in the meristematic areas; RuBisCO small subunit-like promoter element fusion was mainly detected in photosynthetic tissues, with less preponderance in the meristematic region; Ubiquitin-like promoter element fusion was expressed through the gemmae, however poorly co-localized with the expression of *_pro_*Mp*EF1a*:mTurquoise2-N7 reference reporter (Fig. 3A).

Furthermore, to demonstrate the efficiency of MarpoDB for applications in synthetic biology we have performed batch retrieval of promoters elements for all putative transcription factor (TF) genes and designed a library for DNA synthesis with type IIS domesticated versions of these promoter elements (Fig. 3B). We started by performing a BLAST query of all translated MarpoDB CDS sequences against Plant Transcription Factor Database (Jin et al. 2014). Then we retrieved all the promoters that were associated with the CDSs of the putative transcription factors and domesticated them using MarpoDB recode tool. In total, 229 promoter elements were designed for DNA synthesis. A distribution of identified transcription factors is provided in *S1.*

## Discussion

Following the concepts of synthetic biology, we have developed a database framework that compiles genomic data into a gene-centric database of *M. polymorpha* genetic parts. The database will be a useful resource for mining novel genetic components and designing new constructs for plant synthetic biology. We have shown that using predicted promoter elements defined in MarpoDB, we could drive expression patterns of fluorescent reporters *in planta.* We observed distinct gene expression patterns of the fusions, confirming that compact promoter elements are capable of driving gene expression. Whilst these promoter elements were able to drive gene expression, we suspect that native expression is not entirely recapitulated. However, the fact that they are capable of producing distinct expression patterns makes them useful parts for genetic engineering. Furthermore, we have shown that MarpoDB is an efficient tool for extraction of genetic parts for DNA fabrication as common syntax standard parts.

**Fig. 4:**
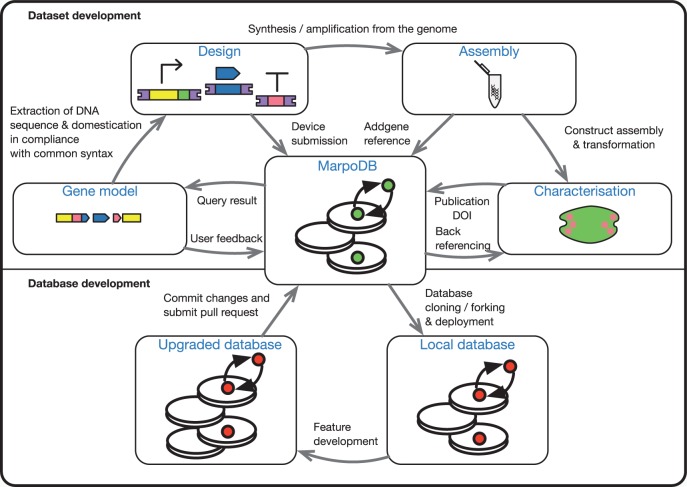
Conceptual map of MarpoDB information flow. Top: dataset development. Results from queries in MarpoDB return the genetic parts required for designing novel genetic constructs, and feedback provided by the users can improve the quality of the annotations. Parts are domesticated for the common syntax for type IIS assembly and produced by synthesis or amplification. Physical parts are assembled and transformed into plants to evaluate their performance. Experimental output is then fed back into MarpoDB to improve the annotations and characterization of the parts, and back referencing to the source data is provided. At the design stage synthetic devices can be submitted to MarpoDB as well as the IDs for the physical location of the assembled plasmid in the form of an Addgene reference. Bottom: database development. MarpoDB can be cloned or forked through git, and local versions of the database can be deployed with custom datasets. Feature developments by users generate upgraded databases and forked repositories can commit changes and submit pull requests to MarpoDB for distributed database development.

The major novelty of MarpoDB is that it stores genetic sequences in a modular fashion, following synthetic biology definitions of standard genetic parts. In combination with the recode tool, this approach allows users to integrate *M. polymorpha* genomic data directly into the common syntax (Fig. 4), implicitly defining modular part level descriptions for higher-level design and assembly. Since genes are defined as part assemblies, adding new synthetic constructs to the database as recombination of existing parts becomes simple. In the future, submission of genetic parts and gene descriptions by users will provide a parts registry for the wider plant synthetic biology community.

We have shown that MarpoDB can be used for batch retrieval of *M. polymorpha* parts by fetching a promoter library of transcription factors and recoding them for type IIS assembly. Recoding of sequences to the common syntax provides the standardization required for generating reusable, shareable libraries of heterologous plant DNA parts. This library will be expanded to include promoter elements and CDSs of other genes of interest and sent for DNA synthesis, thus creating a physical library of *M. polymorpha* parts. Collecting information from individual parts used in different contexts will show how a part performs under different settings. Further characterization of the modules will provide the data for understanding compatibility between the parts, and development of design rules for the construction of synthetic circuits in plants. As the amount of experimental data in *M. polymorpha* increases, part characterization data and topological expression will be included.

Traditionally, we have relied on public centralized genomic resources where community behavior is hidden from the users. Having no pre-publication information about the current state of a research regarding a specific gene, scientists have to compete for the novelty of discovery. Making the community aware of other researchers’ interest on a particular gene, or gene sets, might present opportunities for more comprehensive and collaborative work. This may influence how decisions are made regarding a topic of research, bring scientists with different expertise together to collaborate/apply for grants, or help split workloads between labs. Further, users can give direct feedback on the quality of predictions and annotations and therefore improve the general knowledge regarding a dataset. Following these ideas, we have implemented ‘favouring’ of genes, to allow users to show their interest in a particular gene, as well as saving them on their account for easy access. Along with standard part definitions, we believe this will incentivize researchers to find new ways to collaborate, ensuring consistency between datasets, besides providing a base for decentralized curation.

Finally, the source code of MarpoDB has been made available on GitHub under a MIT license (https://github.com/HaseloffLab/MarpoDB). Our aim is to enable a community-based distributed dataset and database development, where the community improves the state of the art for the available data and new sets of features emerge due to specific needs (Fig. 4). The database itself is agnostic to the genetic sequence source, and basically only requires gene and transcript sequences in fasta format to compile the dataset, whether the sequences originate from high-throughput sequencing or by design. Thus, the database framework can be implemented either for handling genomic data from a gene-centric perspective, or dealing with repositories of genetic constructs. This type of flexibility might encourage people to use local versions of the database for handling libraries of plasmids and genetic parts from their labs and academic centers. We provide the scripts and instructions required for deployment of a local gene-centric database from transcriptome and genome assembly files, and encourage the adoption of the database model for exploring new better ways of dealing with data structures for genetic engineering and synthetic biology.

## Materials and Methods

### Plant methods

#### Plant growth

*M. polymorpha* Cam-1 strain was grown axenically on a 16:8 h day/night cycle in half strength Gamborg’s B5 media + vitamins (Duchefa cat. G0210) with 1% (w/v) agar in 90 mm plates. Individual gemmae were propagated, and after 3 weeks of culture, plants were processed for DNA and RNA extraction.

#### DNA extraction

Two grams of young gametophyte tissue were frozen with liquid nitrogen in a mortar and ground with a pestle into a fine powder (four to five rounds of freezing and crushing). A volume of 10 ml of 65°C pre-warmed cetyl-trimethylammonium bromide (CTAB) extraction buffer (100 mM Tris-HCl pH 8.0, 1.42 M NaCl, 2% (w/v) CTAB, 20 mM EDTA, 2% (w/v) PVP-40, 10 mM ß-Mercaptoethanol, 1 mg ml^−1^ ascorbic acid) were added to the sample in a 50 ml conical Falcon tube and vortexed for 30 s. Next, 10 μl of 100 mg ml^−1^ RNAse A was mixed with solution and incubated at 65°C for 15 min, mixing every 5 min. Ten ml of chloroform:isoamyl alcohol (24:1) was added and mixed by gentle shaking for 30 s. Samples were centrifuged at 4,500 × *g* for 10 min at room temperature and the upper layer was transferred to a new 50 ml conical Falcon tube. Then, 0.7 volume of isopropanol was added to the solution and mixed by inverting the tube four times. The sample was then centrifuged at 4,500 × *g* for 10 min and supernatant was carefully discarded. 70% ethanol was then added and a solid white pellet was re-suspended in the ethanol solution. The pellet was transferred to a new Falcon tube using a cut tip and centrifuged at 13,000 × *g* for 10 min. Supernatant was discarded and pellet was washed with 70% ethanol twice and remaining ethanol was aspirated. The pellet was then left to air-dry for 10 min and re-suspended in 50 μl of Tris-EDTA buffer (10 mM Tris-HCl pH 8.0, 1 mM EDTA). Samples were then processed using the PowerClean Pro DNA Cleanup Kit (MO BIO Laboratories cat. 12997-50), following the manufacturer’s instructions.

#### RNA extraction

Two hundred mg of whole plant tissue (thallus and rhizoids were carefully detached from the agar plate and placed on a 50 mL Falcon tube) were collected from 3-week-old plantlets derived from axenically propagated gemmae and processed for RNA extraction. Total RNA extraction was performed using the RNeasy mini kit (Qiagen cat. 74104), following manufacturer’s instructions. DNAse treatment was conducted with the Turbo DNAse kit (ThermoScientific cat. AM2238) and RNA integrity was assessed using the RNA 6000 nano kit (Agilent cat. 5067-1511) on the Bioanalyzer 2100 machine, according to the manufacturer’s instructions.

#### Agrobacterium-mediated transformation of M. polymorpha

Agrobacterium-mediated transformation was carried out as described previously (Ishizaki et al. 2008) with the following exceptions: half of a archegonia-bearing sporangia (spore-head) was used for each transformation. Dried spore-heads were crushed in a 50 ml Falcon tube with a 15 ml Falcon tube and re-suspended in 1 ml of water per spore-head. Re-suspended spores were filtered through a 40 μm mesh and 1 ml of suspension was aliquoted into a 1.5 ml Eppendorf tube and centrifuged at 13,000 × *g* for 1 min at room temperature. Supernatant was discarded and spores were re-suspended in 1 ml of sterilization solution (one Milton mini-sterilizing tablet dissolved in 25 ml of sterile water (Milton Pharmaceutical UK Company, active ingredient: Sodium dichloroisocyanurate CAS: 2893-78-9: 19.5% w/w) and incubated at room temperature for 20 min at 150 r.p.m. on a shaker. Samples were centrifuged at 13,000 × *g* for 1 min, washed once with sterile water and re-suspended in 100 μl of sterile water per spore-head used. One hundred μl of sterilized spores were inoculated onto half strength Gamborg’s B5 1% (w/v) agar plates and grown under constant fluorescent lighting (50−60 mol photons m-2 s-1) upside down for 5 days until co-cultivation. Sporelings were co-cultivated with previously transformed and induced Agrobacterium GV2260 in 250 ml flasks containing half strength Gamborg’s B5 media supplemented with 5% (w/v) sucrose, 0.1% (w/v) N-Z Amine A (Sigma cat. C7290), 0.03% (w/v) L-Glutamine (Sigma cat. G8540) and 100 μM Acetosyringone (Sigma-Aldrich cat. D134406) for 36 h until washed and plated onto selective media.

### Plasmid construction

Based on the pGreen plasmid (Hellens et al. 2000), a pBRRv7-KpnI plasmid (Fig. S2) containing a *_pro_*Mp*EF1*α:mTurquoise2-N7 cassete, a *_pro_*Mp*UBQ*:TagRFP-T-N7 and a KpnI restriction site upstream of the Venus-N7 sequence was constructed by Gibson Assembly (Gibson et al. 2009). One μg of pBRRv7-KpnI plasmid was subjected to KpnI (NEB cat. R0142S) digestion over-night at 37°C. Digested plasmid was isolated by agarose gel electrophoresis and purified using Qiaquick gel extraction kit (Qiagen cat. 28704) according to the manufacturer’s instructions. Core promoter element sequences were amplified from the *M. polymorpha* genome using Phusion High-Fidelity DNA polymerase (ThermoScientific cat. F530S), isolated by gel electrophoresis and purified using Qiaquick gel extraction kit from Qiagen, according to the manufacturer’s instructions. Purified cut plasmid and promoters elements were assembled by Gibson Assembly. Sequences for the pBRRv7-KpnI plasmid and core promoter elements used for assembly can be found in Figs S3 and S4 respectively.

### Microscopy and image processing

*M. polymorpha* gemma were placed on a microscope slide fitted with a 65 μl Geneframe (ThermoScientific cat. AB-0577), mounted on 10 μg ml^−1^ propidium iodide with a slim coverslip placed on top. Samples were left in the growth chamber and imaged after 12 hours. Imaging was performed on a Leica TCS SP5 confocal microscope with a 10× air objective (HCX PL APO CS 10X 0.4 Dry). Z-stack imaging was performed with 5 μm intervals between slices, collecting fluorescence excited with an Argon laser at 458 nm, 514 nm and with a HeNe 543 nm laser using the sequential imaging mode. Image processing was performed in Fiji for importing LIF files to perform maximum intensity projections of the Z-stacks. Histogram level adaptation was applied in order to remap the intensity values to cover the complete 8-bit range and scale bars were added to the images. Images were then imported to Adobe Photoshop CS6.0 and RGB to CMYK conversion was carried out.

### Bioinformatics

#### Sequencing

DNA sequencing was performed using a 800 bp short-insert library on a Illumina HiSeq 2000 platform with a 100 bp paired-end program on half a sequencing lane. For RNA sequencing, total RNA was used to prepare a cDNA library prepared using the Illumina Plant Ribo-Zero rRNA Removal kit and then sequenced on the Illumina HiSeq 2000 platform with a 100 bp paired-end program on half a sequencing lane.

#### Assembly and filtering

*De novo* genome assembly was performed using the Meraculous 2.0 assembly software (Chapman et al. 2011) using a kmer length of 51, and a *de novo* transcriptome was obtained using the Bridger Assembler (Chang et al. 2015) pipeline. Open-reading frames (ORF) were predicted using the TransDecoder tool (Haas, et al. 2013), keeping ORFs of greater length than 100 amino acids with conserved protein domains from Pfam analysis and homology to *Viridiplantae* protein sequences, extracted from UniprotKB Protein database Release 2016_07 (The Uniprot Consortium, 2014).

#### Annotation

Transcriptome with predicted ORFs was used for downstream analyses. Putative orthologs were identified by querying Uniprot database using BLASTp. InterProScan version 5.16-55.0 (Jones et al. 2014) was run locally to find protein signatures and domains by running the included programs: Hamap, ProDom, PIRSF, PFam, Smart, Gene3D, Coils, ProSiteProfiles, TIGRFAM, Prints, SuperFamily, PrositePatterns, with the retrieve Gene Ontology terms and output to HTML options.

#### Mapping

Mapping of transcripts to genome was performed using Splign (Kapustin et al. 2008) with minimum coverage and identity cut-off values of 99%.

### Server

#### Database

The core of *MarpoDB* is a implemented as a *PostgreSQL* database, with five tables storing sequences of each of the genetic part types (promoter, 5′UTR, CDS, 3′UTR, terminator), a gene table that stores references to one of the parts of each type, and two tables that store either BLASTp or Pfam hits for each of the CDSs.

### Database assembly

#### Web server

The database is accessed via a online web server based on Flask (http://flask.pocoo.org) Python micro-framework. The server uses psycopg (http://initd.org/psycopg/) library for communication with the database and Biopython (Champman & Chang 2000) library for sequence export. The client side exploits Scribl (Miller et al. 2013), a modified HTML5 Canvas based biological charting library for visualizing sequence data, sequence-viewer (https://github.com/calipho-sib/sequence-viewer), a javascript library for sequence viewing, and Clipboard (https://clipboardjs.com) a Javascript library for copying strings into the clipboard.

## Funding

This work was supported by the Chilean Comisión Nacional de Investigación Científica y Tecnológica (CONICYT) [Becas Chile and Cambridge Trust joint Scholarship to B.P.]; the Biotechnology and Biological Sciences Research Council and Engineering and Physical Sciences Research Council Synthetic Biology Research Centre supported by the Research Councils’ Synthetic Biology for Growth Programme [OpenPlant grant No. BB/L014130/1 to J.H.]; and University of Cambridge BBSRC DTP programme [M.D.]

## Supplementary Material

Supplementary Data

## Abbreviations

ID: identification
CDS: coding sequence
ORF: open reading frame
BLAST: basic local alignment search tool
NCBI: National Center for Biotechnology Information
iGEM: International Genetically Engineered Machine Competition
TF: transcription factor
