# Supplementary material for: MarpoDB: An Open Registry for Marchantia Polymorpha Genetic Parts
Source: Plant Cell Physiol. 2017 Jan 27;58(1):e5. doi: 10.1093/pcp/pcw201 (PMC5444569; doi:10.1093/pcp/pcw201)
Supplement: Supplementary Data [file pcw201_Supp.zip › suppl_data/pcp-2016-e-00456-File007.pdf]

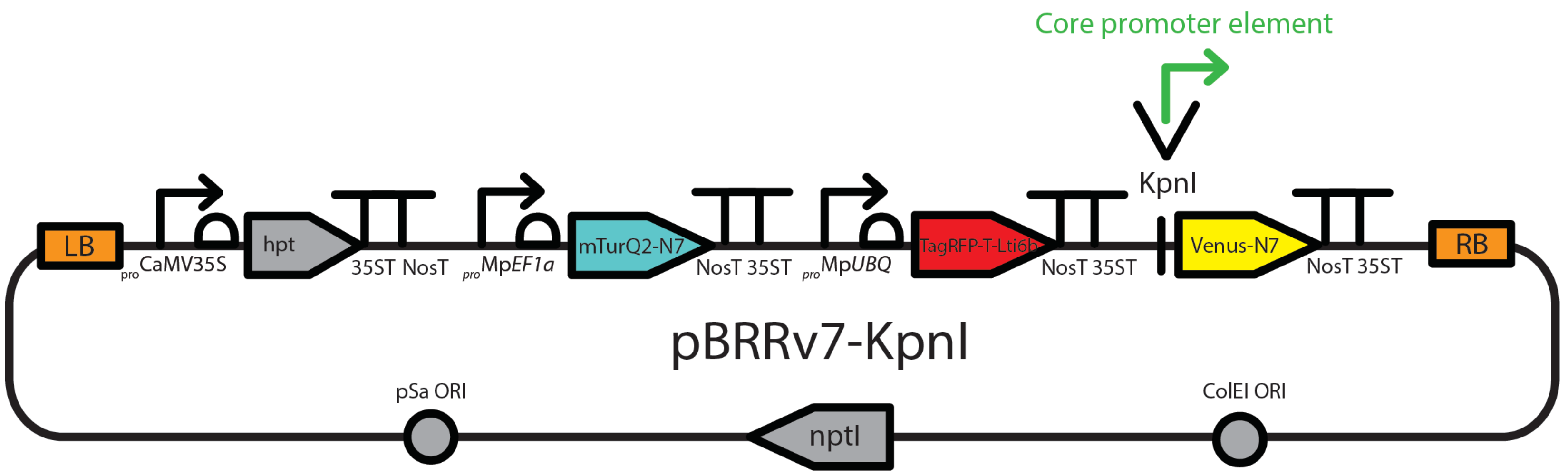

Supplementary figure 2. pBRRv7-KpnI plasmid diagram.
Plasmid elements are shown according to the SBOL standard. KpnI restriction site for core promoter element entry is shown. Plasmid elements include Kanamycin resistance in E. coli, Hygromycin selection for plant selection, constitutive expression of nuclear localised mTurquoise2, nuclear localised Venus and plasma membrane localised TagRFP-T.
