# Supplementary material for: MarpoDB: An Open Registry for Marchantia Polymorpha Genetic Parts
Source: Plant Cell Physiol. 2017 Jan 27;58(1):e5. doi: 10.1093/pcp/pcw201 (PMC5444569; doi:10.1093/pcp/pcw201)
Supplement: Supplementary Data [file pcw201_Supp.zip › suppl_data/pcp-2016-e-00456-File008.pdf]

LB/RB
35S promoter
HygR
Terminators
Promoter
mTurquoise2
N7
Lti6b
TagRFP-T
Venus
nptI (KanR)
KpnI restriction site
ColEI ORI (same as in pGREEN II)
pSa ORI

pBRRv7-KpnI

GATCTTGGCAGGATATATTGTGGTGTAACGTTGTCGTGACTGGGAAAACCCTGGCGTTACCCAACTTAAT
CGCCTTGCAGCACATCCCCCTTTCGCCAGCTGGCGTAATAGCGAAGAGGCCCGCACCGATCGCCCTTC
CCAACAGTTGCGCAGCCTGAATGGCGAATGGCGCGAAATTGTAAACGTTAATGTTATCGTACCCCTACTC
CAAAAATGTCAAAGATACAGTCTCAGAAGACCAAAGGGCTATTGAGACTTTTCAACAAAGGGTAATTTCG
GGAAACCTCCTCGGATTCCATTGCCCAGCTATCTGTCACTTCATCGAAAGGACAGTAGAAAAGGAAGGT
GGCTCCTACAAATGCCATCATTGCGATAAAGGAAAGGCTATCATTCAAGATGCCTCTGCCGACAGTGGTC
CCAAAGATGGACCCCCACCCACGAGGAGCATCGTGGAAAAAGAAGACGTTCCAACCACGTCTTCAAAG
CAAGTGGATTGATGTGACATCTCCACTGACGTAAGGGATGACGCACAATCCCACTATCCTTCGCAAGACC
CTTCCTCTATATAAGGAAGTTCATTTCATTTGGAGAGGACAGCCCAAGCTGATCCCCCCTATGAAAAAGCC
TGAACTCACCGCGACGTCTGTCGAGAAGTTTCTGATCGAAAAGTTCGACAGCGTCTCCGACCTGATGCA
GCTCTCGGAGGGCGAAGAATCTCGTGCTTTCAGCTTCGATGTAGGAGGGCGTGGATATGTCCTGCGGGT
AAATAGCTGCGCCGATGGTTTCTACAAAGATCGTTATGTTTATCGGCACTTTGCATCGGCCGCGCTCCCG
ATTCCGGAAGTGCTTGACATTGGGGAGTTCAGCGAGAGCCTGACCTATTGCATCTCCCGCCGTGCACAG
GGTGTCACGTTGCAAGACCTGCCTGAAACCGAACTGCCCGCTGTTCTTCAGCCGGTCGCGGAGGCTAT
GGATGCTATCGCTGCGGCCGATCTTAGCCAGACGAGCGGGTTCGGCCCATTCGGACCGCAAGGAATCG
GTCAATACACTACATGGCGTGATTTCATATGCGCGATTGCTGATCCCCATGTGTATCACTGGCAAACTGTG
ATGGACGACACCGTCAGTGCGTCCGTCGCGCAGGCTCTCGATGAGCTGATGCTTTGGGCCGAGGACTG
CCCCGAAGTCCGGCACCTCGTGCACGCGGATTTCGGCTCCAACAATGTCCTGACGGACAATGGCCGCA
TAACAGCGGTCATTGACTGGAGCGAGGCGATGTTCGGGGATTCCCAATACGAGGTCGCCAACATCTTCT
TCTGGAGGCCGTGGTTGGCTTGTATGGAGCAGCAGACGCGCTACTTCGAGCGGAGGCATCCGGAGCTT
GCAGGATCGCCACGCCTCCGGGCGTATATGCTCCGCATTGGTCTTGACCAACTCTATCAGAGCTTGGTT
GACGGCAATTTCGATGATGCAGCTTGGGCGCAGGGTCGATGCGACGCAATCGTCCGATCCGGAGCCGG
GACTGTCGGGCGTACACAAATCGCCCGCAGAAGCGCGGCCGTCTGGACCGATGGCTGTGTAGAAGTAC
TCGCCGATAGTGGAAACCGACGCCCCAGCACTCGTCCGAGGGCAAAGGAATAGAGTAGATGCCGACCG
AACAAGAGCTGATTTCGAGAACGCCTCAGCCAGCAACTCGCGCGAGCCTAGCAAGGCAAATGCGAGAG
AACGGCCTTACGCTTGGTGGCACAGTTCTCGTCCACAGTTCGCTAAGCTCGCTCGGCTGGGTCGCGGG
AGGGCCGGTCGCAGTGATTCAGGAATTAATTCGGTACGCTGAAATCACCAGTCTCTCTCTACAAATCTAT
CTCTCTCTATTTTCTCCATAAATAATGTGTGAGTAGTTTCCCGATAAGGGAAATTAGGGTTCTTATAGGGTT
TCGCTCATGTGTTGAGCATATAAGAAACCCTTAGTATGTATTTGTATTTGTAAAATACTTCTATCAATAAAATT
TCTAATTCCTAAAACCAAAATCCAGTACTAAAATCCAGATCGATCACAGGAAACAGCTATGACCATGATTAC
GCCCGGGCTTGACCTGAAGCAGATCAACAACTGGTTCATCAACCAGCGGAAGCGGCACTGGAAGCCAT
CCGAGTAACATCAAAAATGAATCTGCCCGAATTTCCCCGATCGTTCAAACATTTGGCAATAAAGTTTCTTA
AGATTGAATCCTGTTGCCGGTCTTGCGATGATTATCATATAATTTCTGTTGAATTACGTTAAGCATGTAATAA
TTAACATGTAATGCATGACGTTATTTATGAGATGGGTTTTTATGATTAGAGTCCCGCAATTATACATTTAATAC
GCGATAGAAAACAAAATATAGCGCGCAAACTAGGATAAATTATCGCGCGCGGTGTCATCTATGTTACTAGA
TCGGGAATTCATAGTTTGGCCGCGAATTCGCCCTTGATATCCACGTCATTACTCGCATCCATTCTCAGGCT
GTCTCGTCTCGTCTCCAAATGAGTCACACACATTGTTGAGAGACATATCAAAATAACTCAATTGTAAGGTA
AGTTGATAGATTTCCCAGTTAAATGCATTATTTTAACAATATTTGTATCTCAAATTTTCAAAATCATATCAAATT
TGCAGATCTTGATGATGATTCACAACTCTGTCTTCGATTTTTGATTGTTAACGGGTATCGATATTTGGGTAA
CTTAAAAAGAATTTGCTAAAGATCTTAAAAAACAGATTAAAAAAACCTTTCAAAATAGCTAATCCTTTAAATC
CGTTTCACAATATAATGCTCATTGATGATAATCGAAAAGATTCCATTTTAAAGTGCATATAAATTGCTTAATTA
TCTCTTTCTGTCTCGTAATATTTTGTTCTCTCCTATTGGATCGGACCCGCATCTTCTCGATCAGTTCACTAT
ACTAATATTTGGTCAACTTCAAGAGAGATTAGAACTTCGTTCTCCAAAGTGTAAAGGCCGACAAGCAGAAA
AGGAAACTTCAATCAGAAGCCTTCTAGGTTTTCCCGCGTCCCAAAAAGCGAAGGGAGGACGGGGAGGA

CGGTGAGGCCAGAAGAAGAAAGCAACAGTAAAATCTCATGTGATTGCGTGCATGTCATGGATCTATGGGA
TGCTAGTCCACCGTTTGGAAGTATGTGCAGAAGGAGAAAGGCGCGGGAGGCCAAAAGCGACTTCGGCC
ACCTCTCTACTTTCTAGAAAGTTTGCTCGCAGCTTGTAATAAGCGCGGAAAAGGTACTCGAAAAACCGGA
GCAATCGGTGTGGTATCAGCAGTCGGGTTCGCTAAACCTACGTCTTCTTATTTTGGAATGTTAAATTGAAA
GGGCGTGCCGGCCCTAAGCACGGACGATGTGACAGACGGAGGAGTGTGAGCAATTCGGATGACGATG
CTGGCCGGCAGTCACGACAAAGAGTCCGAAGGGGCATGGACAGGGCAATATATAAATGGAAAAGAGGC
TTTGAATATTCCTAACCCTTCGTCGTCGGCCTCCCAACTTGCCCTCGCATGCTGAATTGGCCGCCTATCT
GCGAATGGCCGGCCGCCGACCCTAAGTCGCAATAACTTGAACGGAGCCGATGCAAAGTCCTTATCGAAC
GCTCGTTATTATAAACGGGGCAGACCTAGGTATTTAATTATATCAAAACCCTCCGCAATTTCGCTAATCATT
CCCTAATTTCTTCTACTTTGGCGTCTAGTGCTGTGGTCGCGCTTGCTCTCAAAGCTCGCTGGGAGGCAG
TGGAGCGTCTGGCTTAAAATCGATCGCCGCAACCCTAGAGTTCCTTTCACTTCTTTCTCTCTTGCGTGGA
GAGCATCGACATCGAAGAGCTGCATCTTGTCTTCTGGTATGCGCCTTGTGCCCTCGTCTCTGTACGTCGT
TACGATTGCTCGATATTGTGATTGTCTAGACCTGAAACCCGACTGGAGCTTTTACGTGGTGTCGATTGGG
GCCTCGAGCGAGTGATTTTTTAGGTTCGTTTTAGCCAAGTACATCCCACTTTGGTCAGTCCTGTTATCTCG
GTAAGGTGTTCAGTTCGGTCTCTGGTGCGGAGGTGCCTCGTAAGTTTCCTGCATGATAGATTTGTCTATC
CTTGACTTGAAGAGGAGTCGAAGGTCAGCGAGGTTGGCTGTGTGCTATGGACGTCGGTGCTCCTTGGT
TCCGTGCTTCTATGGGTGTTGCCTCAACAGTATTGATGTGCCTGCAGAAAGGTTGTCACCGTACCATGGT
GAGCAAGGGCGAGGAGCTGTTCACCGGGGTGGTGCCCATCCTGGTCGAGCTGGACGGCGACGTAAAC
GGCCACAAGTTCAGCGTGTCCGGCGAGGGCGAGGGCGATGCCACCTACGGCAAGCTGACCCTGAAGT
TCATCTGCACCACCGGCAAGCTGCCCGTGCCCTGGCCCACCCTCGTGACCACCCTGTCCTGGGGCGT
GCAGTGCTTCGCCCGCTACCCCGACCACATGAAGCAGCACGACTTCTTCAAGTCCGCCATGCCCGAAG
GCTACGTCCAGGAGCGCACCATCTTCTTCAAGGACGACGGCAACTACAAGACCCGCGCCGAGGTGAAG
TTCGAGGGCGACACCCTGGTGAACCGCATCGAGCTGAAGGGCATCGACTTCAAGGAGGACGGCAACAT
CCTGGGGCACAAGCTGGAGTACAACTACTTTAGCGACAACGTCTATATCACCGCCGACAAGCAGAAGAA
CGGCATCAAGGCCAACTTCAAGATCCGCCACAACATCGAGGACGGCGGCGTGCAGCTCGCCGACCACT
ACCAGCAGAACACCCCCATCGGCGACGGCCCCGTGCTGCTGCCCGACAACCACTACCTGAGCACCCA
GTCCAAGCTGAGCAAAGACCCCAACGAGAAGCGCGATCACATGGTCCTGCTGGAGTTCGTGACCGCCG
CCGGGATCACTCTCGGCATGGACGAGCTGTACAAGGCTGCAGCGGCCGAATTCAAGCGTGAAGAGCAA
GCAAGGAAAGCTAAGGTGAACAATGAGAAAAAGACGGAAATAGTGAAACCAGAGAGTTGTAGCAATGAA
GGAGATGTCAAGGATCTGAAAAGAAAGGACTCTGAGGATGGAAACGAGGGTGAGGAAGAAGAAGCTTC
TTCGAAACCGAAAAAGCCAAAAGTTGCTCTTTCTCATCTTCAGGACATTGACGACACAGAAGCTGATCAA
GAAGAAGAGTAAGAGCTCGAATTTCCCCGATCGTTCAAACATTTGGCAATAAAGTTTCTTAAGATTGAATC
CTGTTGCCGGTCTTGCGATGATTATCATATAATTTCTGTTGAATTACGTTAAGCATGTAATAATTAACATGTAA
TGCATGACGTTATTTATGAGATGGGTTTTTATGATTAGAGTCCCGCAATTATACATTTAATACGCGATAGAAA
ACAAAATATAGCGCGCAAACTAGGATAAATTATCGCGCGCGGTGTCATCTATGTTACTAGATCGATCCGTAT
CGATAGCCTCTAGCTAGAGTCGATCGACAAGCTCGAGTTTCTCCATAATAATGTGTGAGTAGTTCCCAGAT
AAGGGAATTAGGGTTCCTATAGGGTTTCGCTCATGTGTTGAGCATATAAGAAACCCTTAGTATGTATTTGTA
TTTGTAAAATACTTCTATCAATAAAATTTCTAATTCCTAAAACCAAAATCCAGTACTAAAATCCAGATGCTGG
GAGTTCGTAGACGGAAACAAACGCAGAATCCAAGCCGTGGATGAGGTTGTATCATTTTTCCCTTTGTTGT
AAGTTTATAAGTTGTTTTTTCGCGGGCATATGTTGCATCAGCCCGCTTAGAGTCAGATGATGATTAGATCC
CAAATATATTATGTGAAATAAATTGATCCAAAAAAGTGTTTGTAAACCTACATCTTAGAAAAAAAAATTATACA
TGTTTTGTCATCCAACTAATAGATCCACTTTTTCACCAATTATAAGGAAAAAGGCGCAAGTAAAATTATCCAT
TTCAAGATGCAAAGCAAATAAACCGTTCACTTCCGCTTCTGCACATATACCATAACATGACTTGCTAGTGG
GCTTGGCGTGAAGCAAATACTTGCTACTATAGTCGGGCTTAGGAACAGCAGAGTATGAATCTGGAATAAA
CCCACGGGGGAGCCCTAAAACCTTTGATTTAGAGAACTTAGGAAGTTCCCAGCTGTAGACTTGCGTAGT
GCGACTTCTGGCCATGCCTACTGTAACCTCCTTGTACCGTTCACGTTCGACCATTTCTCATGATCAAAGA
GTGGATCACATGAAAAAAATGAGCAAATGATAATACATAATATTGTATCGCTGGTTTATTAAGGAGAGTCAA
CTACTGTGCGCTGGAGCGGAGCATCATACGGCGGAGTCGTAGGCAGTTACTGAACCGGAGCTCGTGGT
GACGCTCTTCGTGGTTACTTGAGAAGTTTAACCCATCGTCATCCGGTTGCAGTAACTCACAAGTGGTCTG
TCGGCAGCGGTGACTCACGGCAAGGTCCAGGCGGGAGTCATGATTATCTGCATGTGGTGACGGCATGA
GCTGTCAGCAGCTCGAGGACCCGAAGAGCTATAAAGAACACCCGCTGGTCCTCATCTCCTCATCTCCTC
ATTCTGGTCTGCTTCTTCTTTACCTCACAGCTTCATCTTTCTTTGCTCGTTAAAAGTCTATCTTTCACGTTC
CTTTCTTCATTCGTTTCTACACTTTCTTCTTCGTCCGTTTGTTATTCTGTAGATGGTGTCTAAGGGCGAAGA
GCTGATTAAGGAGAACATGCACATGAAGCTGTACATGGAGGGCACCGTGAACAACCACCACTTCAAGTG
CACATCCGAGGGCGAAGGCAAGCCCTACGAGGGCACCCAGACCATGAGAATCAAGGTGGTCGAGGGC
GGCCCTCTCCCCTTCGCCTTCGACATCCTGGCTACCAGCTTCATGTACGGCAGCAGAACCTTCATCAAC
CACACCCAGGGCATCCCCGATTTCTTTAAGCAGTCCTTCCCTGAGGGCTTCACATGGAGAGAGTCACC
ACATACGAAGACGGGGGCGTGCTGACCGCTACCCAGGACACCAGCCTCCAGGACGGCTGCCTCATCTA
CAACGTCAAGATCAGAGGGGTGAACTTCCCATCCAACGGCCCTGTGATGCAGAAGAAAACACTCGGCT
GGGAGGCCAACACCGAGATGCTGTACCCCGCTGACGGCGGCCTGGAAGGCAGAACCGACATGGCCCT

GAAGCTCGTGGGCGGGGGCCACCTGATCTGCAACTTCAAGACCACATACAGATCCAAGAAACCCGCTAA
GAACCTCAAGATGCCCGGCGTCTACTATGTGGACCACAGACTGGAAAGAATCAAGGAGGCCGACAAAG
AGACCTACGTCGAGCAGCACGAGGTGGCTGTGGCCAGATACTGCGACCTCCCTAGCAAACTGGGGCAC
AAACTTAATGGCATGGACGAGCTGTACAAGTCCGGAGCTGCGGCCGCTGCCGCTGCGGCAGCGGCCG
AATTCAAGCGCTTGAAAATGAGTACAGCCACTTTCGTAGAGATTATTCTTGCTATCATCTTGCCTCCTCTC
GGCGTCTTTCTCAAATTTGGTTGCAAGGTTGAGTTTTGGATATGTTTGATTTTGACGCTGTTTGGTTATCTT
CCCGGAATCCTTTACGCTCTTTATATCATCACCTTTTGAGAGCTCGAATTTCCCCGATCGTTCAAACATTTG
GCAATAAAGTTTCTTAAGATTGAATCCTGTTGCCGGTCTTGCGATGATTATCATATAATTTCTGTTGAATTAC
GTTAAGCATGTAATAATTAACATGTAATGCATGACGTTATTTATGAGATGGGTTTTTATGATTAGAGTCCCGC
AATTATACATTTAATACGCGATAGAAAACAAAATATAGCGCGCAAACTAGGATAAATTATCGCGCGCGGTGT
CATCTATGTTACTAGATCGATCCGTATCGATAGCCTCTAGCTAGAGTCGATCGACAAGCTCGAGTTTCTCC
ATAATAATGTGTGAGTAGTTCCCAGATAAGGGAATTAGGGTTCCTATAGGGTTTCGCTCATGTGTTGAGCA
TATAAGAAACCCTTAGTATGTATTTGTATTTGTAAAATACTTCTATCAATAAAATTTCTAATTCCTAAAACCAAA
ATCCAGTACTAAAATCCAGATGAGCCAACTCCCTTTACAACCTCACTCAAGTCCGTTAGAGGGGAATTAAT
TCACTGGCCGGGTACCATGGTGAGCAAGGGCGAGGAGCTGTTCACCGGGGTGGTGCCCATCCTGGTC
GAGCTGGACGGCGACGTAAACGGCCACAAGTTCAGCGTGTCCGGCGAGGGCGAGGGCGATGCCACCT
ACGGCAAGCTGACCCTGAAGCTGATCTGCACCACCGGCAAGCTGCCCGTGCCCTGGCCCACCCTCGT
GACCACCCTGGGCTACGGCCTGCAGTGCTTCGCCCGCTACCCCGACCACATGAAGCAGCACGACTTCT
TCAAGTCCGCCATGCCCGAAGGCTACGTCCAGGAGCGCACCATCTTCTTCAAGGACGACGGCAACTAC
AAGACCCGCGCCGAGGTGAAGTTCGAGGGCGACACCCTGGTGAACCGCATCGAGCTGAAGGGCATCG
ACTTCAAGGAGGACGGCAACATCCTGGGGCACAAGCTGGAGTACAACTACAACAGCCACAACGTCTATA
TCACCGCCGACAAGCAGAAGAACGGCATCAAGGCCAACTTCAAGATCCGCCACAACATCGAGGACGGC
GGCGTGCAGCTCGCCGACCACTACCAGCAGAACACCCCCATCGGCGACGGCCCCGTGCTGCTGCCCG
ACAACCACTACCTGAGCTACCAGTCCGCCCTGAGCAAAGACCCCAACGAGAAGCGCGATCACATGGTC
CTGCTGGAGTTCGTGACCGCCGCCGGGATCACTCTCGGCATGGACGAGCTGTACATTGCTGCAGCGGC
CGAATTCAAGCGTGAAGAGCAAGCAAGGAAAGCTAAGGTGAACAATGAGAAAAAGACGGAAATAGTGAA
ACCAGAGAGTTGTAGCAATGAAGGAGATGTCAAGGATCTGAAAAGAAAGGACTCTGAGGATGGAAACGA
GGGTGAGGAAGAAGAAGCTTCTTCGAAACCGAAAAAGCCAAAAGTTGCTCTTTCTCATCTTCAGGACATT
GACGACACAGAAGCTGATCAAGAAGAAGAGTAAGAGCTCGAATTTCCCCGATCGTTCAAACATTTGGCAA
TAAAGTTTCTTAAGATTGAATCCTGTTGCCGGTCTTGCGATGATTATCATATAATTTCTGTTGAATTACGTTA
AGCATGTAATAATTAACATGTAATGCATGACGTTATTTATGAGATGGGTTTTTATGATTAGAGTCCCGCAATT
ATACATTTAATACGCGATAGAAAACAAAATATAGCGCGCAAACTAGGATAAATTATCGCGCGCGGTGTCATC
TATGTTACTAGATCGATCCGTATCGATAGCCTCTAGCTAGAGTCGATCGACAAGCTCGAGTTTCTCCATAAT
AATGTGTGAGTAGTTCCCAGATAAGGGAATTAGGGTTCCTATAGGGTTTCGCTCATGTGTTGAGCATATAA
GAAACCCTTAGTATGTATTTGTATTTGTAAAATACTTCTATCAATAAAATTTCTAATTCCTAAAACCAAAATCC
AGTACTAAAATCCAGATCTCGTTCGCTGCCACCTAAGAATACTCTACGGTCACATACCCCTGGCGTTAGG
GATAACGCAGGAAAGAACATGAAGGCCTTGACAGGATATATTGGCGGGTAAACTAAGTCGCTGTATGTGT
TTGTTTGAGATCTCATGTGAGCAAAAGGCCAGCAAAAGGCCAGGAACCGTAAAAAGGCCGCGTTGCTGG
CGTTTTTCCATAGGCTCCGCCCCCCTGACGAGCATCACAAAAATCGACGCTCAAGTCAGAGGTGGCGAA
ACCCGACAGGACTATAAAGATACCAGGCGTTTCCCCCTGGAAGCTCCCTCGTGCGCTCTCCTGTTCCGA
CCCTGCCGCTTACCGGATACCTGTCCGCCTTTCTCCCTTCGGGAAGCGTGGCGCTTTCTCATAGCTCAC
GCTGTAGGTATCTCAGTTCGGTGTAGGTCGTTCGCTCCAAGCTGGGCTGTGTGCACGAACCCCCCGTTC
AGCCCGACCGCTGCGCCTTATCCGGTAACTATCGTCTTGAGTCCAACCCGGTAAGACACGACTTATCGC
CACTGGCAGCAGCCACTGGTAACAGGATTAGCAGAGCGAGGTATGTAGGCGGTGCTACAGAGTTCTTGA
AGTGGTGGCCTAACTACGGCTACACTAGAAGAACAGTATTTGGTATCTGCGCTCTGCTGAAGCCAGTTAC
CTTCGGAAGAAGAGTTGGTAGCTCTTGATCCGGCAAACAAACCACCGCTGGTAGCGGTGGTTTTTTTGT
TTGCAAGCAGCAGATTACGCGCAGAAAAAAAGGATCTCAAGAAGATCCTTTGATCTTTTCTACGGGGTCT
GACGCTCAGTGGAACGAAAACTCACGTTAAGGGATTTTGGTCATGAGATTATCAAAAAGGATCTTCACCT
AGATCCTTTTAAATTAAAAATGAAGTTTTAAATCAATCTAAAGTATATATGTGTAACATTGGTCTAGTGATTAG
AAAAACTCATCGAGCATCAAATGAAACTGCAATTTATTCATATCAGGATTATCAATACCATATTTTTGAAAAA
GCCGTTTCTGTAATGAAGGAGAAAACTCACCGAGGCAGTTCCATAGGATGGCAAGATCCTGGTATCGGT
CTGCGATTCCGACTCGTCCAACATCAATACAACCTATTAATTTCCCCTCGTCAAAAATAAGGTTATCAAGTG
AGAAATCACCATGAGTGACGACTGAATCCGGTGAGAATGGCAAAAGTTTATGCATTTCTTTCCAGACTTG
TTCAACAGGCCAGCCATTACGCTCGTCATCAAAATCACTCGCATCAACCAAACCGTTATTCATTCGTGATT
GCGCCTGAGCGAGACGAAATACGCGATCGCTGTTAAAAGGACAATTACAAACAGGAATCGAATGCAACC
GGCGCAGGAACACTGCCAGCGCATCAACAATATTTTCACCTGAATCAGGATATTCTTCTAATACCTGGAAT
GCTGTTTTCCCTGGGATCGCAGTGGTGAGTAACCATGCATCATCAGGAGTACGGATAAAATGCTTGATGG
TCGGAAGAGGCATAAATTCCGTCAGCCAGTTTAGTCTGACCATCTCATCTGTAACAACATTGGCAACGCT
ACCTTTGCCATGTTTCAGAAACAACTCTGGCGCATCGGGCTTCCCATACAATCGGTAGATTGTCGCACCT
GATTGCCCGACATTATCGCGAGCCCATTTATACCCATATAAATCAGCATCCATGTTGGAATTTAATCGCGGC

CTTGAGCAAGACGTTTCCCGTTGAATATGGCTCATAACACCCCTTGTATTACTGTTTATGTAAGCAGACAG
TTTTATTGTTCATGATGATATATTTTTATCTTGTGCAATGTAACATCAGAGATTTTGAGACACAACGTGGCTT
TGTTGAATAAATCGAACTTTTGCTGAGTTGAAGGATCAGATCACGCATCTTCCCGACAACGCAGACCGTT
CCGTGGCAAAGCAAAAGTTCAAAATCACCAACTGGTCCACCTACAACAAAGCTCTCATCAACCGTGGCT
CCCTCACTTTCTGGCTGGATGATGGGGCGATTCAGGCGATCCCCATCCAACAGCCCGCCGTCGAGCGG
GCTTTTTTATCCCCGGAAGCCTGTGGATAGAGGGTAGTTATCCACGTGAAACCGCTAATGCCCCGCAAAG
CCTTGATTCACGGGGCTTTCCGGCCCGCTCCAAAAACTATCCACGTGAAATCGCTAATCAGGGTACGTG
AAATCGCTAATCGGAGTACGTGAAATCGCTAATAAGGTCACGTGAAATCGCTAATCAAAAAGGCACGTGA
GAACGCTAATAGCCCTTTCAGATCAACAGCTTGCAAACACCCCTCGCTCCGGCAAGTAGTTACAGCAAGT
AGTATGTTCAATTAGCTTTTCAATTATGAATATATATATCAATTATTGGTCGCCCTTGGCTTGTGGACAATGC
GCTACGCGCACCGGCTCCGCCCGTGGACAACCGCAAGCGGTTGCCCACCGTCGAGCGCCAGCGCCTT
TGCCCACAACCCGGCGGCCGGCCGCAACAGATCGTTTTATAAATTTTTTTTTTTGAAAAAGAAAAAGCCC
GAAAGGCGGCAACCTCTCGGGCTTCTGGATTTCCGATCCCCGGAATTA

Supplementary figure 3. Plasmid pBRRv7-KpnI annotated sequence file.
Sequence file for plasmid pBRRv7-KpnI is shown with genetic elements highlighted in different colours. Colour legend is shown on top.
