# Supplementary material for: MarpoDB: An Open Registry for Marchantia Polymorpha Genetic Parts
Source: Plant Cell Physiol. 2017 Jan 27;58(1):e5. doi: 10.1093/pcp/pcw201 (PMC5444569; doi:10.1093/pcp/pcw201)
Supplement: Supplementary Data [file pcw201_Supp.zip › suppl_data/pcp-2016-e-00456-File009.pdf]

>Alpha-tubulin like
GGCATTTCACATTTCATAACTGGAGAGTTGTCCAGAGCTTGAACGGATCAAAGTGGACTAGGAGCCTGTT
ATGTGGATATCGAACTGACGCAGTTGAAGTCTGTAAGGCTACCACATCCGTACACCAGCCTGTCCTGACG
CCCGTCAGGCTTTCAGCAACGTGGGGTCGACGATCACGAAGGGGGTATCATTTAGTCCAGTAACGGAAC
GAAGTTGTGTGGCGTCGTCCTACGTGTCCGGTCTCGTATGCCAATATTCTCCCAATGACCGGTTAAGTTT
GAATGTCTAGCTGCGGAGAGAAAATAGAGGCGATCGTCGACCTCCACCCGCCCGTCGACCCCAACATC
GTAGGGCTCCCATGAGACATTTGACGGGATCAGGAATAATGGATGGAATGGTGTGGACCCTACTAATAGA
AGGAAAGCCAGATTGCTACGCGTTTTATGCCATGTGAATGTGACTAGGCTTGCATGTGTAAGGTGAGCAC
TGACTTGATATTTCGGTGGAATAGCTCCTGGTCCAGCTTGCAACTCCTGCTTGCACCCCTCACTTTCCCA
TTGCATATTCCTCCCCTTTCAAATCCTACATATCCCTTTCTTGCTGGCACAACCTCAAAGCGTCACCCTTT
CCCTTGTTTTTTCTTGATTTATAAATGTCAGCAAACTGGGCTACCCAAGAATTAAAAGGCGAAAGTGCAAA
CTTTTGGAATTCTTTCGGGTCTCGTGTCACATTTTTTTCATCTGACCTGAACATGATAAATCCACAGTAAAA
AAAACTGGAAGTAGGTTTTTTTTTGTGAGAAAATTGCCACCTTATTTCCAATTATTTCAAGTTTTGTATCCAA
ATTTTCTATGATGTTTAAATCCAAAGTGGATTTAAGATGTAAGAAGCCGTGCATTTTTTCGGATGGAAATTC
ACTTACAGCTTGAAAAATAAAGAATCTGATTTAAAGCTTGGGTTATTTGCGGATGATACGATTGGTGATACA
AAGAGATTTATTGCATGTACCGCGCTCTTATGAAAGGTTAGGGAGCTCGCTAAATGGTAATTCGTCGATTA
GGGCTTCATGACTGATCACGATCTCGTTGTGGGAACTGGAAACGACTTTTCTTCAGGGAATTACACTTTA
AGATGATTTGGGATATTTACGAACTTGTTAACCGTATGAGTGTGTTCTGATACTGCAAGTACAAATCGGATA
GGGGCTTTGTTCACTCATGAATCGTACTTAGAAATAGATGACTGGGATAGAAGGTTAAGCAGGACATGTT
CCTAGCCTTTTTGCGCTAATAAATTTCATTTGAGCGAATATAAGCCGATAAAAAGTGGCGGCAGACGGTGT
TTGAGGCATAACTTAATCCGTGGAATTAAAAGTGTCAGAAGCGAGAGTACAGTAATGACATTGTTCTTAAC
ACAAGTCGGAGAAGGTTCCATGTGTTCTTGTAACGACGCGGAAAGCAGGTGCGGCAAAAGCTGGCGAA
AGTGGCGAGAGAGTGAGACAAAGGGAGATATTATTCCAAGGGCAGCCCGAGAAATCATCAACGGGCGG
GGTCAACGAGGAGCCGCTCTCTTCACCCCGCGTGGCGCGGCGCTGGTCCGCTCCCCGTCTTCTCCCG
TCGTCTGCCCTGCCCGGCGTTGCCCTCGCCCGGCCCAGCCCGGCCCGCGCTGCGCTGCATTGCCCTG
GCCCGGTCGTCCTTTGCCTTCCCTTCTCGTGCTGCGTTGGCTGCGAGACATCGCTTACTAGTCGCGAAT
GTTGTAGATCTTCATCTCTGCTTGTGCCGTTGTTGTTCTCTGTGCGCTTGTGCTGCGCATCCGTTGCCTT
GCCTGTCCTCCCTCCTCGCGCAGCGTTCGTGAGCGTCCTCGACACGCTCGCCGGCTGCCTTTCATTAC
TTGTTCTCTCGCCTCCCATCTCTCGTCTCTCGCCTTCTGTTCTCTTCCTCAATTTTCCGATCTCTTATACCA
TCGATCGATCTGATCAGGAGGAAGCAGGCACGACGAGA

>RuBisCO like
TGTAATAGTCTTTCGGTCTGTCCACTTCTAATCTTTCGGTACAGGCTGACCTGCCCTCCGGTCCATAACAG
AGAACGAATCTACTTCAACGATTGCTCCTAGACAAATTAGCACACGGTTCTAAGATACATGATTCGGATTC
TGCTCATGCACTTTGAGCACTATAGCTGTGTGTAGTATAGTGATACCTGTCCGGACAACTGGAGAAACTTA
GGGTTTCTCCAGTTGTCCGGACATATATGTATTATAGTAGGTACTAGGTAATAGCTTCATTAGCCATTCCTG
ACAAAAAAAAAAAACAATTCTAGGTTGTTTAGAAAGTCCATTTTACAAAATATATAAATGATCCAATTTAGAAG
AAATTACTAACTTACTTGACGCTGAAGATGAATACTATCGAATAGCTACTGGAAAATGCTTAGCATTAGATC
AAAATAGTCAAGAAGAAAAAATTACTCGACAAGATTTTGTAGCTACTGCACGGGAAAATAATATTTTTTAAA
AAAAACTACTTGTATAATTTTAAAATGATTTAAGAAGATTGTTGTGATTGAATTAGTACATTTTTAACCAATAA
AATAAATAAATTATCTATACCAAAATAAAAATATTATTGAAATTTTAGAATACGCTAACCATCCATTTAAAAAAA
ATTGAATGAATAAGTGTTGAGTTGAAAAAAAATGTTTATTTTGAAATACTCATCTTTAAATATTATATTTATGAT
AATTATTTCATCAGTTAAGAGAACAATATTAAAAAGTTTGGTTCTTTCAAGTGATGAAATTCATTGCTACCAG
AATTTAAAATTTTGAAAATACTGTAGAATCAATCTTCAATATAGTCAAACAAAGATGTAGATTTTTATGAAATT
GGATTTGAATTCAATAATTTATTATTTGATATATATTATCCAGCTAAATAAGTGATCACTTACTTCCAGATCAAG
CATCCAAATTTTAACTTGTAGAATCCTAAAATGAATGTCTCTACAACTAGAATTATTTGTAGAATTTCCAAAG
TAGCGTTATGGAAACTATAACATATCTATCGAATAAATAACAGTCTAACAAAATTGTTGCAAAAAACTCTAAG
ATAGATATAATTCATAACGAAAGGAGCCAAACAAGGTTACGCTTCAGTAACATTACAACGGTACATATGTCA
TAAATCACTTAAGATATTTAATGATATATTTAAACATACAACAGGAATGTATGAGCAATCATCCTTTACTGCAA
TTGAGATTTAATAGATTTCAATGATTACTGAGTTCTAAAGGGCAATGTACACACTTTGCTTAAAATGTAGTTC
AAAATAAATTAACGATCGTATATTTAATTTTTGCACATAATTACAAGCATTTTGAAGTTGCTGTCTATGGTTAC
ATGAGAAGTCAACCCAAGTGAATTCTTGCATATTTATAAATTTATATATTTTTATTTTTTCTTTTCCACTCGAA
ATGACTATCATTAATCATTGACCAGTACACATTACATATCTATTTTATCGTTAATTGGTAACACTCATGAGCGA
ATAATGAAATAAAAATCTTGAACGGTCCAACCATAAGATAAGATAGAAGATATTCAGTTGAAAGATTTAGTG
CAAATATATGAATGGATATGAAAAACTCATGCATCACACACGAAATCGATATCTACTATCAATTTATCCACAT
GACGTAAGATGCAGCGAAAGCGCTTGTACTTTATGCAGCAAAGCACTGTGATAACAGCATTGCAGTGCAC
AACGCCATGGAGAGCTGGACGGCTGATGTCACGCGGTGAACTCAAATCAACGTCGTCGATACGCACAAT
CTCATCACGAATATCGATCGATCTTGAGTTCTGTGGTTCTCGTGATCAGCGCACTCCATCTTCAACCTGTG
GCTGTCCAGCCTCATCTGCTCCTCTCCAGCCTGTTCTATCCTTTCGTACAAGGACTACTTAATACCGGGG
TCCACGCCTAGTTAGATCCCACAGCACACACTTTGCATTC

>UBQ like
CAACAGAGAGAAAATATACTGTACTTTGTTGTTTACGTAGCTTCATCTTCTTTGACCATGTCGGCTGTGGC
GATTCACGGAAGATTCACGAATAAAATTGATGGCAAGAACCGAACGTGCCAGAACTTACTTTATCACTTTT
CCAATATAGAAGAATAAAAGAAATATAGCCAACAAAGCAAAAGAACGAAAAAGATCTTTGCGAACGAAGGG
TCTGGAGACGCTGAGTGGTCGCAGCAACCTTTGAGCCATCGGTCAGCCGTAGACGGCACAGTCGGTCT
TCTCACTTCCGGTACAAGCAACTCATCATGCCCGAACGGCTATCCATCTCTGACCGTCTGACGTCTGTCC
CGTTTACAACATCGGGACGACGGGAGACCCCGTCGGGAGTTCCTCTTTTGTCGTATATGTGGAACCTTT
GGTAAAGTTTAAGCTCCTTTAACTGAAAAGTCGATTGATCTCCTGTAACAGCCACGTGGCAACCGGCTAC
TGTAATCTGCATTGTCGAAAAAAGGACGAAGACATTGATTTTTGAGGAAAGTTTAAAATGATATAGAGTTGA
TCGAGAGATTCATGCCCGTTTTGAAACAGTGAGTAAGATATTAAGAATTATAATTTGTTATCTACTTGTTGCC
ACGTTGGTGACACGCTGTACCAGTACAGGTGCCGGTTGAATACTATTTTAGCATGGGACGTACTTTTGAC
AGACCGTCGCTTGATTTTGGTCCACATTCAATCAACTACATCTGAGTTTGCTTCATTAGCAGTGGCCGGTA
GCGCCACCGGCCACCAATTTCAATGTTTCAACTAAGTCTCGTTCGGGGTTTTCTAAATTATCTAGAGTTTC
GAGCTAAATCTACAGCGGGAATAAGTCACGACAGTTTAGAATAAGAGTCACTACAATTAGTAGAGCGTTTA
GGGTCTAGATTACTTGTATTTGAGCTACAATATTGTTTTTTTGGCGTCATTGTCTGCATCAAACGTGACACT
CAGAGACGTGGATGAGGTTGTATCATTTTTCCCTTTGTTGTAAGTTTATAAGTTGTTTTTTCGCGGGCATAT
GTTGCATCAGCCCGCTTAGAGTCAGATGATGATTAGATCCCAAATATATTATGTGAAATAAATTGATCCAAA
AAAGTGTTTGTAAACCTACATCTTAGAAAAAAAAATTATACATGTTTTGTCATCCAACTAATAGATCCACTTT
TTCACCAATTATAAGGAAAAAGGCGCAAGTAAAATTATCCATTTCAAGATGCAAAGCAAATAAACCGTTCAC
TTCCGCTTCTGCACATATACCATAACATGACTTGCTAGTGGGCTTGGCGTGAAGCAAATACTTGCTACTAT
AGTCGGGCTTAGGAACAGCAGAGTATGAATCTGGAATAAACCCACGGGGGAGCCCTAAAACCTTTGATTT
AGAGAACTTAGGAAGTTCCCAGCTGTAGACTTGCGTAGTGCGACTTCTGGCCATGCCTACTGTAACCTCC
TTGTACCGTTCACGTTCGACCATTTCTCATGATCAAAGAGTGGATCACATGAAAAAAATGAGCAAATGATA
ATACATAATATTGTATCGCTGGTTTATTAAGGAGAGTCAACTACTGTGCGCTGGAGCGGAGCATCATACGG
CGGAGTCGTAGGCAGTTACTGAACCGGAGCTCGTGGTGACGCTCTTCGTGGTTACTTGAGAAGTTTAAC
CCATCGTCATCCGGTTGCAGTAACTCACAAGTGGTCTGTCGGCAGCGGTGACTCACGGCAAGGTCCAG
GCGGGAGTCATGATTATCTGCATGTGGTGACGGCATGAGCTGTCAGCAGCTCGAGGACCCGAAGAGCTA
TAAAGAACACCCGCTGGTCCTCATCTCCTCATCTCCTCATTCTGGTCTGCTTCTTCTTTACCTCACAGCTT
CATCTTTCTTTGCTCGTTAAAAGTCTATCTTTCACGTTCCTTTCTTCATTCGTTTCTACACTTTCTTCTTCGT
CCGTTTGTTATTCTGTAG

Supplementary figure 4. Core promoter element sequences for tested promoter fusions.
Sequences for M. polymorpha Alpha-tubulin like, Rubisco like and UBQ like promoters are shown in FASTA format. Core promoter elements including 5'UTRs were identified and cloned into pBRRv7-KpnI for driving the expression of nuclear localised Venus-N7.
